# The Effect of Medical Cooperation in the CKD Patients: 10-Year Multicenter Cohort Study

**DOI:** 10.3390/jpm13040582

**Published:** 2023-03-26

**Authors:** Yasuhiro Onishi, Haruhito A. Uchida, Yohei Maeshima, Yuka Okuyama, Nozomu Otaka, Haruyo Ujike, Keiko Tanaka, Hidemi Takeuchi, Kenji Tsuji, Masashi Kitagawa, Katsuyuki Tanabe, Hiroshi Morinaga, Masaru Kinomura, Shinji Kitamura, Hitoshi Sugiyama, Kosuke Ota, Keisuke Maruyama, Makoto Hiramatsu, Yoshiyuki Oshiro, Shigeru Morioka, Keiichi Takiue, Kazuyoshi Omori, Masaki Fukushima, Naoyuki Gamou, Hiroshi Hirata, Ryosuke Sato, Hirofumi Makino, Jun Wada

**Affiliations:** 1 Department of Nephrology, Rheumatology, Endocrinology and Metabolism, Okayama University Faculty of Medicine, Dentistry and Pharmaceutical Sciences, Okayama 700-8558, Japan; 2 Department of Comprehensive Therapy for Chronic Kidney Disease, Okayama University Faculty of Medicine, Dentistry and Pharmaceutical Sciences, Okayama 700-8558, Japan; 3 Department of Chronic Kidney Disease and Cardiovascular Disease, Okayama University Faculty of Medicine, Dentistry and Pharmaceutical Sciences, Okayama 700-8558, Japan; 4 Advanced Medical Engineering Research Institute, University of Hyogo, Himeji 670-0914, Japan; 5 Olba Healthcare Holdings Inc., Okayama 700-0907, Japan; 6 Japanese Red Cross Society Himeji Hospital, Himeji 670-8540, Japan; 7 Kagawa Prefectural Central Hospital, Takamatsu 760-8557, Japan; 8 National Hospital Organization Okayama Medical Center, Okayama 701-1154, Japan; 9 Okayama Saiseikai General Hospital, Okayama 700-8511, Japan; 10 Department of Medical Care Work, Kawasaki College of Allied Health Professions, Okayama 700-8505, Japan; 11 Okayama Saiseikai Outpatient Center Hospital, Okayama 700-0013, Japan; 12 Kawasaki Medical School General Medical Center, Okayama 700-8505, Japan; 13 Okayama Central Hospital, Okayama 700-0017, Japan; 14 Okayama City Hospital, Okayama 700-0962, Japan; 15 Shigei Medical Research Hospital, Okayama 701-0202, Japan; 16 Japanese Red Cross Okayama Hospital, Okayama 700-0941, Japan; 17 Akebono Clinic, Okayama 702-8056, Japan; 18 Sato Clinic, Okayama 700-0864, Japan; 19 Okayama University, Okayama 700-8530, Japan

**Keywords:** chronic kidney disease (CKD), medical cooperation, patient care team, OCKD-NET

## Abstract

Introduction: While chronic kidney disease (CKD) is one of the most important contributors to mortality from non-communicable diseases, the number of nephrologists is limited worldwide. Medical cooperation is a system of cooperation between primary care physicians and nephrological institutions, consisting of nephrologists and multidisciplinary care teams. Although it has been reported that multidisciplinary care teams contribute to the prevention of worsening renal functions and cardiovascular events, there are few studies on the effect of a medical cooperation system. Methods: We aimed to evaluate the effect of medical cooperation on all-cause mortality and renal prognosis in patients with CKD. One hundred and sixty-eight patients who visited the one hundred and sixty-three clinics and seven general hospitals of Okayama city were recruited between December 2009 and September 2016, and one hundred twenty-three patients were classified into a medical cooperation group. The outcome was defined as the incidence of all-cause mortality, or renal composite outcome (end-stage renal disease or 50% eGFR decline). We evaluated the effects on renal composite outcome and pre-ESRD mortality while incorporating the competing risk for the alternate outcome into a Fine–Gray subdistribution hazard model. Results: The medical cooperation group had more patients with glomerulonephritis (35.0% vs. 2.2%) and less nephrosclerosis (35.0% vs. 64.5%) than the primary care group. Throughout the follow-up period of 5.59 ± 2.78 years, 23 participants (13.7%) died, 41 participants (24.4%) reached 50% decline in eGFR, and 37 participants (22.0%) developed end-stage renal disease (ESRD). All-cause mortality was significantly reduced by medical cooperation (sHR 0.297, 95% CI 0.105–0.835, *p* = 0.021). However, there was a significant association between medical cooperation and CKD progression (sHR 3.069, 95% CI 1.225–7.687, *p* = 0.017). Conclusion: We evaluated mortality and ESRD using a CKD cohort with a long-term observation period and concluded that medical cooperation might be expected to influence the quality of medical care in the patients with CKD.

## 1. Introduction

Chronic kidney disease (CKD) is one of the most important contributors to morbidity and mortality from non-communicable diseases worldwide [1], with global prevalence as high as 9–13% [2,3]. However, the number of nephrologists is limited in all countries [4]; in addition, even in Japan, where there are a relatively large number of nephrologists (59.27 per 1 million population), some problems have been pointed out: renal function and urinary findings are not routinely measured in CKD patients, and standard medication such as renin-angiotensin system (RAS) inhibitors and erythropoiesis stimulating agent (ESA) preparations are not given to CKD patients [5]. Approximately 12.9–14.6% of the Japanese adult population was estimated to have CKD in 2015 [6]. Since Japan is one of the super-aging countries, the number of CKD patients is likely to increase further due to the aging of the population [7].

While most countries report a shortage of nephrologists, fewer countries report a shortage of primary care physicians [4]. Since education of primary care physicians has been shown to reduce the risk of decline in kidney function and death [8,9], the Kidney Disease: Improving Global Outcomes (KDIGO) Controversies Conference suggested the need for education of primary care physicians [10]. In Japan, medical cooperation between primary care physicians and nephrologists has been recognized as one of the models to educate and support primary care physicians in CKD care [11,12]. Medical cooperation is a system of collaboration between primary care physicians and nephrological institutions, consisting of nephrologists and multidisciplinary care teams [13]. Although it has been reported that multidisciplinary care teams contribute to the prevention of worsening renal functions and cardiovascular events [14,15,16,17], there are few studies on the effect of medical cooperation between primary care physicians and nephrologists. Okayama City is one of the regions with the most advanced medical cooperation for CKD. We have been organizing the cooperation model for the past 10 years and developing a network of primary care physicians and nephrologists, called the Okayama city CKD Network (OCKD-NET) [18]. The impact of this cooperation model on mortality risk and the renal prognosis has not yet been explored, and we aim to clarify its long-term relevance.

## 2. Materials and Methods

### 2.1. Study Subjects

We recruited prospective outpatients who visited primary care physicians and/or nephrologists in 7 general hospitals between December 2009 and September 2016. The enrollment institutions consisted of 162 clinics and 7 general hospitals participating in the OCKD-NET. The inclusion criteria of this study were as follows: (i) 18 years of age or older; (ii) the presence of CKD; defined as the presence of either of the conditions listed below lasting for more than 3 months: (a) with markers of kidney damage, such as proteinuria, hematuria, etc., (b) decreased eGFR less than 60 mL/min/1.73 m^2^ [19,20]. The exclusion criteria of this study were as follows: (i) withdrawal of consent; (ii) follow-up period of less than 1 year.

This study was conducted in accordance with the principles of the Declaration of Helsinki, and the protocol was approved by the Ethics Committee of Okayama University Hospital (authorization number: K1506-045). All study participants had the opportunity to opt-out of this study by visiting the website or the enrollment institutions.

### 2.2. Variables and Data Sources

Patients provided demographic and clinical information at enrolment. Variables included age, gender, causes of renal disease, hypertension, and diabetes mellites (DM). Risk factors reported previously for patients to develop end-stage renal disease (ESRD) were evaluated; blood pressure, dip-stick test for proteinuria, urine protein-to-creatinine ratio, serum creatinine (sCr), total cholesterol, triglycerides, high-density lipoprotein (HDL) cholesterol, low-density lipoprotein (LDL) cholesterol, uric acid, and hemoglobin. The dip-stick test for proteinuria was semi-quantified as (−), (±), (1+), (2+), (3+), or (4+). The eGFR was calculated using the following estimation equation for Japanese patients with CKD (eGFR (mL/min/1.73 m^2^) = 194 × age^−0.287^ × sCr^−1.094^ × (0.739 for women)) [21]. CKD G staging was classified according to the Kidney Disease Improving Global Outcomes (KDIGO) guideline [22], with an eGFR ≥ 90, 60–89, 45–59, 30–44, 15–29, and <15 mL/min/1.73 m^2^ classified as G1, G2, G3a, G3b, G4, and G5, respectively. Proteinuria was classified into 3 categories: KDIGO A1 category of negative proteinuria, dip-stick test (−), or urinary protein excretion <0.15 g/gCr; A2 category of trace proteinuria, dip-stick test (±), or urinary protein excretion 0.15–0.49 g/gCr; and A3 category of positive, dip-stick test ≥ (1+), or urinary protein excretion ≥0.5 g/gCr [23]. The target value of blood pressure (BP) was defined as systolic BP less than 130 mmHg and diastolic BP less than 80 mmHg [20].

The medical cooperation of participants was defined by the collaboration of primary care physicians and nephrologists. The nephrologists provided expert opinion at least once a year, depending on the patients’ situation, and mutually discussed with primary care physicians in writing, as well as providing multidisciplinary support and referrals to other departments as needed. The participants were assigned to a medical cooperation group or primary care group at baseline. If the patient is seen only by a primary care physician, the patient will be assigned to the primary care group and provided appropriate treatment by one physician.

### 2.3. Outcomes

The primary outcome for this analysis was defined as incidence of all-cause mortality, ESRD, or CKD progression. ESRD was defined as the initiation of hemodialysis, peritoneal dialysis, or kidney transplantation. CKD progression was defined as a decline of at least 50% in the eGFR from baseline. The secondary outcomes included individual endpoints comprising the primary outcome; all-cause mortality, or renal composite outcome (ESRD or CKD progression). The follow-up evaluation of patients who did not develop each outcome was censored at the last clinical visit (i.e., the patient transferred to another medical institution), or the end of the study, as appropriate.

### 2.4. Statistical Analysis

Data were summarized as percentages, means ± standard deviation (SD), or medians [interquartile range; IQR]. Categorical variables were analyzed with Fisher’s exact test and continuous variables were compared using the *t*-test or Mann–Whitney U test, as appropriate. To compare the achievement rates of blood pressure control targets between the baseline and the final observation, we used the McNemar test. Cumulative survivals were estimated with Kaplan–Meier survival curves and compared by log-rank test. Analysis for outcomes was assessed using the Fine–Gray model because all-cause mortality is a competing risk for ESRD [24]. The Fine–Gray model was implemented in the STATA “stcrreg” module. HR was first calculated in the crude analysis; medical cooperation or not. Subsequent models were adjusted for confounding factors at baseline; male and age equal to or greater than 65 years old (model 1); model 1 plus CKD stage G and A according to KDIGO classification (model 2); *p* values less than 0.05 were considered to be significant. All statistical analyses were performed using Stata software (version 17.0; Stata Corporation, College Station, TX, USA).

## 3. Results

### 3.1. Clinical Characteristics of the Participants

**Figure 1:**
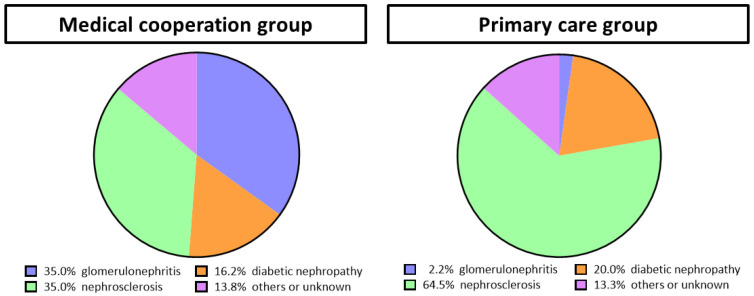
The causes of kidney disease in the medical cooperation group and the primary care group. All patients were classified as one of the following renal diseases based on renal biopsy or clinical diagnosis by the attending physician: glomerulonephritis, diabetic nephropathy, nephrosclerosis, other renal diseases, or unknown. The medical cooperation group tended to have more glomerulonephritis, while the primary care group tended to have more nephrosclerosis.

**Figure 2:**
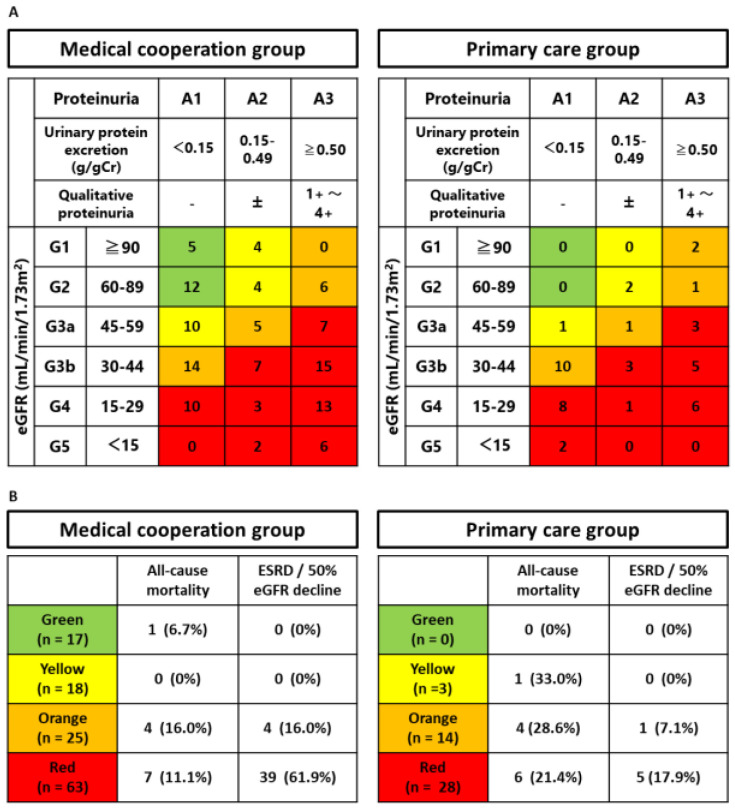
The association between CKD stage and medical cooperation. (**A**) The CKD stages of the participants are shown by the group as KDIGO 2012 heatmap at baseline. (**B**) The incidence of the outcomes is shown by KDIGO 2012 heatmap.

We recruited 168 participants with a follow-up period of 5.59 ± 2.78 years. In total, 123 participants were classified into the medical cooperation group and 45 participants were assigned into the primary care group (Supplementary Figure S1). The patients in the medical cooperation group visited nephrologists a median of three times per year, or every 4 months, in 9 out of 10 years (Supplementary Figure S2). Nutritional guidance was provided by a registered dietitian during the follow-up period in 56% of the medical cooperation group patients, with a frequency of a median of 0.5 (0.3–1.1) sessions per year. Baseline characteristics divided by medical cooperation are presented in Table 1. In comparison with the primary care group, the medical cooperation group demonstrated a younger mean age (61.7 ± 17.3 vs. 76.0 ± 9.6 years old, *p* < 0.001), a larger proportion of men (74 (60.2%) vs. 17 (37.8%), *p* = 0.010), and a higher eGFR (41.6 [IQR: 27.7–61.8] vs. 33.0 [IQR: 26.9–42.6] mL/min/1.73 m^2^, *p* = 0.024). The rates of hypertension and diabetes, proteinuria, lipids, and uric acid were similar between the two groups. As shown in Figure 1, there was more glomerulonephritis in the medical cooperation group (43, 35.0% vs. 1, 2.2%) and more nephrosclerosis in the primary care group (43, 35.0% vs. 29, 64.5%). In the OCKD-NET, it seems that the patients were referred to nephrologists according to the recommendation of the Japanese Society of Nephrology [18], which may have influenced the profile of patients enrolled. The CKD stages of the participants were shown as KDIGO 2012 heatmap in Figure 2A. Since the patients suspected of having glomerulonephritis are referred to nephrologists even if proteinuria is not high, there were more patients with A1 to 2 in the medical cooperation group.

### 3.2. Outcomes

**Figure 3:**
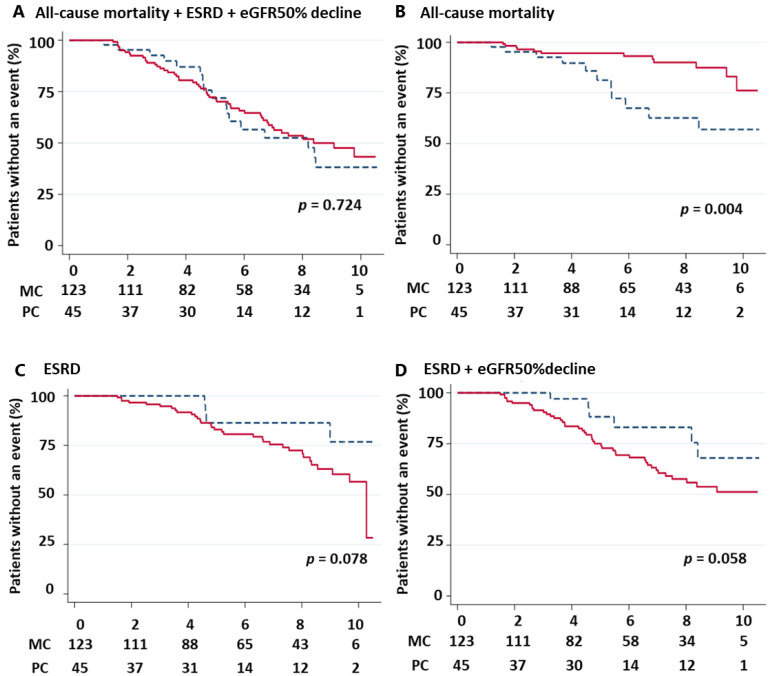
Kaplan–Meier analyses for primary and secondary outcomes based on baseline medical cooperation. The red solid line represents the event-free time of the medical cooperation group. The blue dash line represents the event-free time of the primary care group. The survival curves were compared in medical cooperation for (**A**) All-cause mortality + ESRD + eGFR50% decline, (**B**) All-cause mortality, (**C**) ESRD, (**D**) CKD progression (ESRD + eGFR 50% decline). Abbreviation: MC, medical cooperation. PC, primary care; ESRD, end-stage renal disease.

A total of 41 participants (24.4%) reached 50% decline in eGFR, and 37 participants (22.0%) developed ESRD. Twenty-three participants (13.7%) died with the following causes: eight cardiovascular, three infectious, five cancer, and seven unknown. The incidence of the outcomes and the cumulative survival rates were shown in Figure 2B and Figure 3, respectively. The incidence of ESRD or 50% decline in eGFR was higher in the medical cooperation group (43, 35.0% vs. 6, 13.3%; *p* = 0.006). However, the difference was not significant in the log-rank test because it took longer to reach the event, probably due to prolonged renal prognosis (*p* = 0.058). The incidence of death was higher in the primary care group (12, 9.8% vs. 11, 24.4%; *p* = 0.014), with most deaths occurring in the heatmap orange-red group. The results of log-rank analyses similarly showed a higher risk of death in the primary care group (*p* = 0.004). Both death and CKD progression were competing for risks, and the significance of the difference disappeared when the composite outcome was used (*p* = 0.724). To assess the association between medical cooperation and outcomes, Fine–Gray analyses were performed (Table 2). After multivariate adjustments for major confounding factors, all-cause mortality was significantly reduced by medical cooperation (sHR 0.297, 95% CI 0.105–0.835, *p* = 0.021). Although the crude analysis showed no significant association between medical cooperation and CKD progression (sHR 2.737, 95% CI 0.990–7.567, *p* = 0.052), after full adjustment, there was a significant association for CKD progression (sHR 3.069, 95% CI 1.225–7.687, *p* = 0.017) or ESRD only (sHR 2.795, 95% CI 1.080–7.231, *p* = 0.034).

### 3.3. The Effect of Medical Cooperation on the Blood Pressure Control

**Figure 4:**
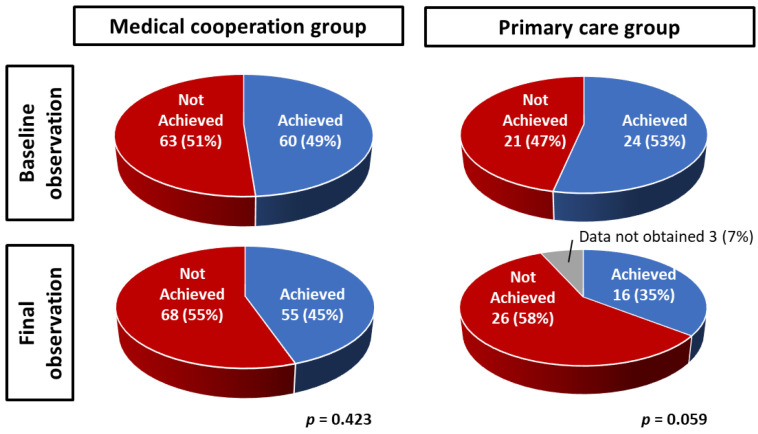
Effect of medical cooperation on achievement of blood pressure control. The target value of blood pressure was defined as systolic BP less than 130 mmHg and diastolic BP less than 80 mmHg [18]. At baseline, the medical cooperation group and the primary care group had similar rates of achieving blood pressure control targets.

To examine the effect of medical cooperation, we evaluated the achievement of blood pressure control targets at baseline and at final observation (Figure 4). The baseline observations showed the achievement rates for the medical cooperation group and the primary care group were similar at 49% and 53%, respectively (*p* = 0.883). At the final observation, the achievement rates of blood pressure control targets were 45% in the medical cooperation group and 35% in the primary care group; there was no difference in the medical collaboration group (*p* = 0.423), but there was a trend toward lower achievement rate in the primary care group (*p* = 0.059).

## 4. Discussion

We evaluated mortality and ESRD using a CKD cohort with a long-term observation period and showed that medical cooperation might be associated with a reduced risk of all-cause mortality in the Japanese patients with CKD. Conversely, medical cooperation was associated with CKD progression in this cohort.

Medical cooperation consists of a patient-centered care team of primary care physicians and nephrologists. Recently, medical cooperation within a region or hospital has been attracting attention [25,26,27]. While the medical resource of primary care physicians is relatively abundant, nephrologists in general hospitals have a multidisciplinary network including additional specialists, nurses, and a variety of other healthcare professionals. The usefulness of multidisciplinary care in chronic diseases, such as resistant hypertension and diabetes mellitus, has been suggested since the early 2000s [28,29,30,31]. CKD is known to worsen in association with such non-communicable diseases, so the effects of multidisciplinary care on CKD have been investigated. The Canadian population-based, propensity score-matched retrospective cohort study showed that the elderly patients with CKD treated in the multidisciplinary care clinics had a 50% lower risk of all-cause mortality than the patients treated in other clinics [32]. The cluster-randomized study, FROM-J, showed that the CKD practice facilitation program by multidisciplinary care for primary care physicians reduced cardiovascular events [17]. The meta-analysis of 10,284 CKD patients from 21 randomized controlled trials and cohorts (including Asian populations) provides robust evidence that multidisciplinary care decreases the odds ratio of all-cause mortality by 0.67 (95% CI: 0.51–0.88, *p* < 0.01) [33]. In these studies, interventions were carried out by the specialists comprising the multidisciplinary care team. However, in the present study, mortality was improved only by interventions judged necessary by the nephrologists, suggesting that the interventions via medical cooperation were efficient. With regards to the control of blood pressure, while there was a trend toward lower achievement rates in the primary care group, it was maintained in the medical cooperation group. In addition to multidisciplinary care, interventions by multiple physicians may have led to an avoidance of clinical inertia.

The effect of medical cooperation and multidisciplinary care on the renal prognosis has been investigated for each CKD stage. In the CKD patients who were progressing to ESRD, the benefit of early referral to the nephrologist, such as lower mortality and shorter duration of hospitalization, has been noted and recommended [20,34]. The meta-analysis of 63,387 CKD patients from 40 cohorts showed that early referral reduced the 34% risk ratio for mortality at five years [35]. The KDIGO guideline recommends referral to a nephrologist for CKD patients with stage G4 or proteinuria > 0.5 g/gCr/albuminuria > 300 mg/gCr [22]. The guideline of the Japanese Society of Nephrology recommends referral to a nephrologist for the patients with CKD at stage G3b or higher, or for early-stage CKD patients at stage G3a with proteinuria > 0.15 g/gCr or age < 40 years [20]. In such patients with stage 3/4 CKD, the effect of multidisciplinary and nephrologist care has been shown to improve all-cause mortality, but the renal protective effect is controversial. An observational study reported that multidisciplinary team care for stage 3 CKD resulted in a slower decline in eGFR at mean follow-up of 2 years [36], but an RCT of intervention by nephrologists and nurse practitioners in CKD patients with a median eGFR of 42 mL/min/1.73 m^2^ for 20 months found no difference in the rate of eGFR decline [37]. Because of the limited follow-up period in these studies, hard outcomes such as ESRD have not been evaluated. The present study is different in that it assessed hard outcomes with an observation period of more than twice as long as those previously reported. In addition, because the causes of kidney disease of the enrolled patients were not limited, active nephritis was enrolled in the medical cooperation group, which may have affected the renal prognosis.

This study showed that medical cooperation improved mortality but did not show the improvement in renal prognosis. To improve the quality of interventions, two aspects of medical cooperation need to be strengthened: On-the-job training of physicians, and the promotion of patients‘ health behaviors from multidisciplinary professionals. KDIGO proposed education for primary care physicians, including interactive workshops and guideline symposia [10]. The OCKD-NET, a network of primary care physicians and nephrologists in Okayama City, held regular symposia to discuss cases and introduce guidelines for more than 10 years. In addition, since the same patients are treated by nephrologists and primary care physicians, medical cooperation is expected to have an indirect educational effect. The establishment of such a medical cooperation network may gradually improve the practices of participating physicians through on-the-job training. Additionally, multidisciplinary care is known to improve the patients’ positive health behaviors [38]. The key to personalized medicine is to clarify in which populations there are behavioral changes due to medical cooperation and which interventions are responsible for those changed behaviors. Since it was difficult to measure the results of behavioral changes in this study, we plan to examine these points in the next study, which is currently underway.

Several limitations of our study also warrant mention. First, since this is an observational study, we were unable to adjust for unmeasurable confounders such as the practice behavior of each primary care physician. Second, reflecting the real world, the allocation was not randomized and biased by the influence of each primary physician’s decision on medical cooperation, and there is a case bias between the medical cooperation group and the primary care group. To avoid this problem, we adjusted for age, gender, CKD stage, and proteinuria by multivariate analysis. Due to the limited number of participants in the primary care group, it is difficult to analyze after propensity score matching. Caution is needed in interpreting the effect of age on mortality. In addition, difficult-to-treat patient populations, i.e., patients with hepatorenal syndrome and psychiatric disorders, are more likely to be eligible for medical cooperation. These patients could not be excluded or adjusted for in the multivariate analysis. The results reflect actual clinical practice and support the good external validity of this study. Third, the content of medical cooperation is not standardized. Medical cooperation is conducted between primary care physicians and nephrologists. However, this also reflects actual clinical practice, and mortality rates could be improved by nephrologists providing personalized care to the patients with primary care physicians. Fourth, outcomes may not be tracked sufficiently, but they are inquired about by the secretariat and tracked as much as possible via telephone or other means, by individual attending physicians. Deaths after the introduction of dialysis could not be partially assessed because of the change in family practice. Fifth, since many patients of the primary care group are treated by primary care physicians alone and have limited routine testing, data on the control of anemia, diabetes, and mineral and bone disorder in CKD (CKD-MBD), etc., are missing. For example, management of CKD-MBD reduces vascular calcification and mortality [39,40,41], but we were unable to analyze the relationship between medical coordination and mechanism. Sixth, we were not able to assess whether urgent hospitalizations and cardiovascular events were more common in the primary care group. The number of cardiovascular deaths is limited and not statistically different (medical cooperation: 4% vs. primary care: 6.7%, *p* = 0.443), but the deaths of unknown cause, including sudden death, tended to be more common in the primary care group (medical cooperation: 2.4% vs. primary care: 8.9%, *p* = 0.084). The risk for cardiovascular death is notably increased in the patient with CKD [42], and we plan to elucidate the effects of medical cooperation on mortality improvement in the next study. Seventh, there was a higher proportion of glomerulonephritis in the medical cooperation group, which may have affected renal outcomes. In Japan, patients with glomerulonephritis are often treated by nephrologists, and the number of cases in the primary care group was limited, which reflects real world scenarios.

In conclusion, we evaluated the long-term effect of medical cooperation between primary care physicians and nephrologists in CKD patients. We found that medical cooperation might be expected to change the quality of medical care through on-the-job training of primary care physicians and the improvement of patients’ health behaviors. We need to further validate medical cooperation for CKD patients by evaluating which changes were beneficial and linking them to personalized medicine in the future.

## Figures and Tables

**Table 1:** Characteristics of the study population.

	Medical Cooperation Group (N = 123)		Primary Care Group (N = 45)		*p* Value
Follow-up, months	69.9 ± 33.8		62.7 ± 34.2		0.223
Age, years	61.7 ± 17.3		76.0 ± 9.6		<0.001
Male, n (%)	74	(60.2)	17	(37.8)	0.010
Hypertension, n (%)	88	(71.5)	35	(77.8)	0.419
Diabetes mellitus, n (%)	34	(27.6)	11	(24.4)	0.679
Systolic blood pressure, mmHg	130.8 ± 21.1		127.6 ± 15.2		0.648
Diastolic blood pressure, mmHg	74.6 ± 13.8		69.4 ± 11.3		0.031
Serum creatinine, mg/dL	1.27 (0.92–1.71)		1.28 (1.06–1.56)		0.742
eGFR, mL/min/1.73 m^2^	41.6 (27.7–61.8)		33.0 (26.9–42.6)		0.024
Qualitative proteinuria, −/±/1+/2+/3+/4+	52/16/26/20/7/2		21/6/11/4/3/0		0.799
Urinary protein excretion, g/gCr	0.25 (0.09–1.01)		0.56 (0.11–0.94)		0.870
Total cholesterol, mg/dL	193 (164–221)		208 (199–217)		0.506
Triglyceride, mg/dL	143 (93–203)		118 (70–165)		0.516
HDL-C, mg/dL	54 (44–70)		73 (58–88)		0.240
LDL-C, mg/dL	110 (92–136)		115 (94–135)		0.977
Uric acid, mg/dL	6.7 (5.5–7.4)		4.3 (3.5–5.0)		0.054
Hemoglobin, g/dL	12.8 (10.9–14.0)		11.6 (10.7–13.4)		0.107

Values were expressed by percentage, mean ± SD, or median (interquartile range). Abbreviations: eGFR, estimated glomerular filtration rate; HDL-C, high-density lipoprotein cholesterol; LDL-C, low-density lipoprotein cholesterol.

**Table 2:** Competing-risk model of variables associated with the cumulative incidence of all-cause mortality or ESRD/50% eGFR decline.

	All-Cause Mortality			ESRD + eGFR50% Decline		
	Subhazard Ratio	95% CI	*p* Value	Subhazard Ratio	95% CI	*p* Value
Crude (Medical Cooperation)	0.243	0.102–0.578	0.001	2.737	0.990–7.567	0.052
Model 1: male + 75 years old or older	0.293	0.105–0.817	0.019	3.437	1.094–10.794	0.034
Model 2: model 1 + CKD stage (CKDG, CKDA)	0.297	0.105–0.835	0.021	3.069	1.225–7.687	0.017

The associations with all-cause mortality or renal composite events and medical cooperation are shown. Analysis for outcomes was assessed using Fine–Gray subdistribution hazard model. Abbreviations: 95% CI, 95% confidence interval.

## Data Availability

Not applicable.

## Supplementary Materials

The following are available online at https://www.mdpi.com/article/10.3390/jpm13040582/s1, Figure S1: The flow diagram, Figure S2: Frequency of nephrology visits for the patients in the medical cooperation group by survey year.
