# Supplementary material for: The Effect of Medical Cooperation in the CKD Patients: 10-Year Multicenter Cohort Study
Source: J Pers Med. 2023 Mar 26;13(4):582. doi: 10.3390/jpm13040582 (PMC10142789; doi:10.3390/jpm13040582)
Supplement: Supplementary file 1 [file jpm-13-00582-s001.zip › jpm-2220237-supplementary.pdf]

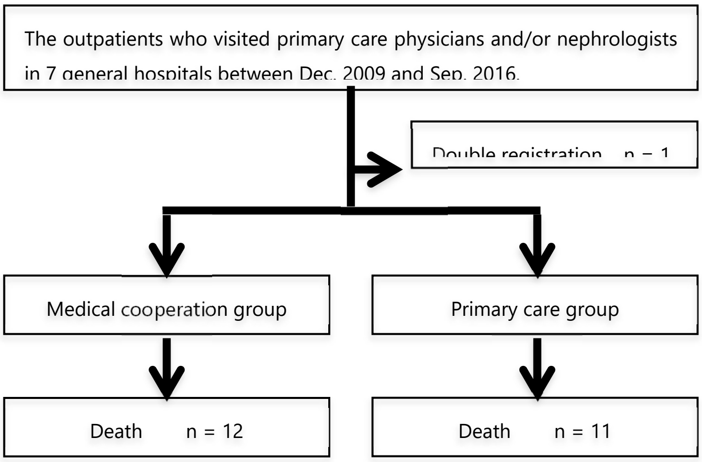

Supplementary Figure S1. The flow diagram

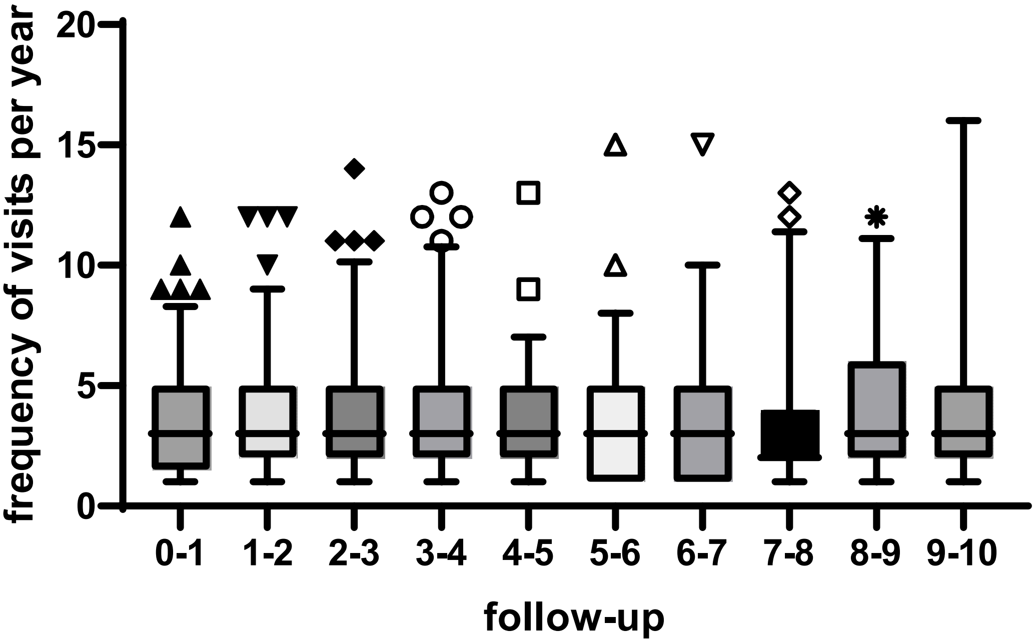

Supplementary Figure S2. Frequency of nephrology visits for the patients in the medical cooperation group by survey year
